# An Intelligent Grey Wolf Optimizer Algorithm for Distributed Compressed Sensing

**DOI:** 10.1155/2018/1723191

**Published:** 2018-01-31

**Authors:** Haiqiang Liu, Gang Hua, Hongsheng Yin, Yonggang Xu

**Affiliations:** China University of Mining and Technology, Xuzhou 221116, China

## Abstract

Distributed Compressed Sensing (DCS) is an important research area of compressed sensing (CS). This paper aims at solving the Distributed Compressed Sensing (DCS) problem based on mixed support model. In solving this problem, the previous proposed greedy pursuit algorithms easily fall into suboptimal solutions. In this paper, an intelligent grey wolf optimizer (GWO) algorithm called DCS-GWO is proposed by combining GWO and *q*-thresholding algorithm. In DCS-GWO, the grey wolves' positions are initialized by using the *q*-thresholding algorithm and updated by using the idea of GWO. Inheriting the global search ability of GWO, DCS-GWO is efficient in finding global optimum solution. The simulation results illustrate that DCS-GWO has better recovery performance than previous greedy pursuit algorithms at the expense of computational complexity.

## 1. Introduction

Compressed sensing (CS) [1, 2] is a new signal sampling theory which has broken through the limit of Nyquist sampling theorem. If there are no more than *k* nonzero entries in the signal **x** ∈ *R*^*n*^, **x** is called a sparse signal and the sparsity of **x** is *k*. If **x** is sparse, it can be recovered from much fewer samples. We can get the measurement signal **y** = Φ**x** by projecting **x** onto the measurement matrix Φ ∈ *R*^*m*×*n*^, where *m* ≪ *n*. Because *m* < *n*, it is an NP-hard problem to recover **x** from **y**. However, if *k* < *m* < *n* and Φ satisfies the Restrict Isometry Property (RIP) with order *k*, **x** can be perfectly recovered. Gaussian random matrix [1], partial Fourier matrix [3], Bernoulli random matrix [2], and so on can be used as measurement matrix. Greedy pursuit algorithms [4–7], *l*_1_ minimization algorithms [8–10], and intelligent optimal algorithms [11–13] are proposed to recover **x** from **y**.

CS theory just exploits intrasignal correlation, which makes it not efficient in dealing with multiple signals. An expanded version of CS, Distributed Compressed Sensing (DCS) [14, 15], which can exploit not only intrasignal correlation but also intersignal correlation is proposed. With proper joint recovery algorithms, the measurement number needed in DCS can be further reduced. In this paper, we call the problem of jointly recovering signals as DCS problem. Several joint sparse models (JSM) and corresponding joint recovery algorithms are proposed to solve the DCS problem. One-Step Greedy Algorithm (OSGA) [15] is proposed to solve the DCS problem based on JSM-1. Greedy pursuit algorithms, including Simultaneous Orthogonal Matching Pursuit (SOMP) [15], Simultaneous Iterative Hard Thresholding (SIHT) [16], and Simultaneous Hard Thresholding Pursuit (SHTP) [16] are proposed to solve the DCS problem based on JSM-2. In [17, 18], two intelligent optimization algorithms based on particle swarm optimization and simulated annealing are proposed to solve the DCS problem based on JSM-2. However, as our analysis in Section 2.1, JSM-1 and JSM-2 are stringent on the description of signal correlation, which makes them reflect less intersignal and intrasignal correlations. In [19], JSM-3 is proposed. As a generalization of JSM-1 and JSM-2, it can reflect more intersignal and intrasignal correlations. This paper focuses on solving the DCS problem based on JSM-3. We notice that Joint Subspace Pursuit (Joint-SP) [20], Joint Orthogonal Matching Pursuit (Joint-OMP) [20], Sparsity Adaptive Matching Pursuit for DCS (DCS-SAMP) [21], and Forward-Backward Pursuit for DCS (DCS-FBP) [22] are proposed to solve the DCS problem based on JSM-3. However, as greedy pursuit algorithms, they easily fall into suboptimal solutions.

GWO algorithm [23] is proposed by Mirjalili et al. in 2014, which simulates the hunting behavior and leadership hierarchy of grey wolves. As top apex predators, grey wolves normally live and hunt in a pack which includes 5–12 wolves. As an optimization algorithm, GWO has probability to accept a less optimal solution, which makes it avoid being stuck in local optimal solutions. Because of this, GWO draws much attention in solving some optimization problems and NP hard problems. Kumar et al. [24] apply GWO in system reliability optimization. Mirjalili [25], Hassanin et al. [26], and Sánchez [27] adopt GWO to train Artificial Neural Network (ANN). Muangkote et al. [28] propose an improved GWO algorithm and apply it in training *q*-Gaussian Radial Basis Functional-link nets (qRBFLNs) neural networks. Li et al. [29] propose a modified discrete GWO algorithm to solve the image segmentation problem. Emary et al. [30] propose a binary GWO algorithm and use it to select the optimal feature for the purpose of classification. In these areas, GWO performs better or comparable to other prevailing nature-inspired optimization algorithms [31–35].

In solving the DCS problem based on JSM-3, greedy pursuit algorithms easily fall into suboptimal solutions. From the above analysis of GWO, we know that it is a recently proposed global search optimization algorithm. Its performance is superior or comparable to other prevailing algorithms in solving some optimization problems. As illustrated in Section 3, the DCS problem based on JSM-3 can be modeled as an optimization problem. These reasons motivate us to exploit GWO to solve the DCS problem based on JSM-3.

In this paper, an intelligent grey wolf optimizer (GWO) [23] algorithm called DCS-GWO is proposed to solve the DCS problem based on JSM-3. DCS-GWO is essentially a GWO algorithm, the grey wolves' positions are initialized by using the *q*-thresholding algorithm [36] and updated by using the strategy of GWO. Inheriting the global search ability of GWO, DCS-GWO has better recovery performance than previous greedy pursuit algorithms at the expense of computational complexity.

The remainder of this paper is organized as follows. In Section 2, we introduce related background knowledge, including DCS model, joint sparse models (JSM), grey wolf optimizer (GWO) algorithm, and *q*-thresholding algorithm. In Section 3, we introduce the DCS-GWO algorithm. In Section 4, we provide the simulation results. Conclusions are stated in Section 5.

We use the following notations in this paper. Lowercase bold-face denotes a vector. Uppercase bold-face denotes a matrix. For the vector **x** ∈ *R*^*n*^, ‖**x**‖_*q*_  (*q* > 1) denotes the *l*_*q*_ norm of **x**. If ‖**x**‖_0_ ≤ *k* < *n*, **x** is called sparse signal and the sparsity is *k*. *T*≜{1 ≤ *i* ≤ *n*∣**x**(*i*) ≠ 0} denotes the support set of **x**. For the matrix Φ ∈ *R*^*m*×*n*^, Φ_:,*j*_ denotes the *j*th column of Φ and Φ_*i*,:_ denotes the *i*th row of Φ. For the set *S* = {1,2,…, *n*}, |*S*| denotes the cardinality of *S*. *G*⊆*S* denotes that *G* is a subset of *S*. Φ_:,*G*_ denotes the matrix composed of the columns {Φ_:,*j*_}_*j*∈*G*_. Φ_*G*,:_ denotes the matrix composed of the rows {Φ_*i*,:_}_*i*∈*G*_. Φ^*T*^ denote the transpose matrix of the matrix Φ. Φ^†^ = (Φ^*T*^Φ)^−1^Φ^*T*^ denotes the pseudo-inverse matrix of Φ.

## 2. Background Knowledge

In this part, we introduce related background knowledge of this paper. The DCS model is introduced in Section 2.1. Joint sparse models are introduced in Section 2.2. Grey wolf optimizer is introduced in Section 2.3. At last, the *q*-thresholding algorithm is introduced in Section 2.4.

### 2.1. DCS Model

Suppose that there are *J* signals **x**_*j*_ ∈ *R*^*n*^ which are sparse and have the sparsity *k*^*j*^ < *n*, where *j* ∈ {1,2,…, *J*}. They are individually measured by the measurement matrix Φ ∈ *R*^*m*×*n*^ as [14, 21]. That is,

(1) $$\begin{array}{c} y_{j}=\Phi \times x_{j},\;j\in \left\{1,2,\ldots ,J\right\}. \end{array}$$

Without the consideration of noise, the measurement process can be denoted as

(2) $$\begin{array}{c} Y=\Phi X, \end{array}$$

where **X** = [**x**_1_, **x**_2_,…, **x**_*J*_] ∈ *R*^*n*×*J*^ denotes the joint signal and **Y** = [**y**_1_, **y**_2_,…, **y**_*J*_] ∈ *R*^*m*×*J*^ denotes the measurement signal. The DCS problem is to recover **X** from **Y** jointly by exploiting intersignal and intrasignal correlations. For the matrix **X** ∈ *R*^*n*×*J*^, the set of indices corresponding to nonzero rows of **X** is the joint support set of **X**, which can be denoted as *R*(**X**)≜{1 ≤ *i* ≤ *n*∣**X**_*i*,:_ ≠ 0}. If there are no more than *K* nonzero rows in **X**, **X** is called jointly sparse and the joint sparsity is *K*.

### 2.2. Joint Sparse Models

The JSM reflects the intersignal correlations and intrasignal correlations. There are mainly three JSMs.

*(1) JSM-1. *JSM-1 is called sparse common support and innovation model [14, 15]. JSM-1 can be written as

(3) $$\begin{array}{c} x_{j}=z+z_{j}, \end{array}$$

where *j* ∈ {1,2,…, *J*}. **z** is the common component shared by all signals. **z**_*j*_ is the innovation component for each signal. The sparsity of **z** is *K*_**z**_. The sparsity of **z**_*j*_ is *K*_*j*_.

*(2) JSM-2. *JSM-2 is called sparse common support model [14, 15]. JSM-2 can be written as

(4) $$\begin{array}{c} x_{j}=z_{j}, \end{array}$$

where *j* ∈ {1,2,…, *J*}. For each signal, the support set of **z**_*j*_ is the same, but the coefficients are individual. The sparsity of **z**_*j*_ is *K*_*j*_.

*(3) JSM-3. *JSM-3 is called mixed support model [19, 20]. JSM-3 has common component **c**_*j*_ and innovation component **z**_*j*_. For each signal, the support set of **c**_*j*_ is the same, but the nonzero coefficients are individual. For each signal, the innovation component is completely independent, not only the coefficients but also the support set. JSM-3 can be written as

(5) $$\begin{array}{c} x_{j}=c_{j}+z_{j}, \end{array}$$

where *j* ∈ {1,2,…, *J*}. The sparsity of **c**_*j*_ is *K*_*c*_. The sparsity of **z**_*j*_ is *K*_*j*_. Obviously, JSM-3 is a generalization of JSM-1 and JSM-2. If **z**_*j*_ = 0, JSM-3 reduces to JSM-2. If both the coefficients and support set of **c**_*j*_ are the same for each signal, JSM-3 reduces to JSM-1. JSM-3 is less stringent in describing signal correlations, so, it can reflect more signal correlations. Our algorithm is proposed to solve the DCS problem based on JSM-3.

### 2.3. Grey Wolf Optimizer

Grey wolf optimizer (GWO) [31] algorithm is a recently proposed intelligent optimization algorithm. As apex predators, grey wolves own special leadership hierarchy and hunting mechanism. Grey wolves are divided into four categories, alpha (*α*), beta (*β*), delta (*δ*), and omega (*ω*). *α* is the leader which makes decision to hunt, rest, forward, or stop. *β* assists *α* make decision and reinforces *α*'s commands. *δ* executes the decision and manages *ω* wolves which are the lowest ranking of grey wolves.

Before hunting, the grey wolves firstly encircle the prey. The distance between the wolf and prey is computed by using (6). The wolf's position is updated by using (7).

(6) d=c·hpt−ht

(7) ht+1=hpt−ad,

where *t* denotes the current iteration, **h**_*p*_ denotes the prey's position vector, **a** and **c** are two coefficient vectors, and **h** denotes a grey wolf's position vector. The coefficient vectors **a** and **c** are determined as follows:

(8) a=2vγ2−vc=2γ1,

where **γ**_1_, **γ**_2_ are random vectors between 0 and 1 and the vector **v** decreases from 2 to 0 linearly in the iteration course.

After the process of encircling the prey, the hunting is led by *α*, *β*, and *δ*. All wolves' positions are updated according to the positions of *α*, *β*, and *δ*. Firstly, the distances between a wolf and the best three wolves are computed by using (9). Then, the position of the wolf is updated by using (10) and (11).

(9) dα=c1hαt−ht,dβ=c2hβt−ht,dδ=c3hδt−ht

(10) h1=hα−a1dα,h2=hβ−a2dβ,h3=hδ−a3dδ

(11) hpt+1=h1+h2+h33

After all wolves' positions are updated, the process of hunting the prey goes to the next iteration in which the new best three solutions are generated. The iteration repeats until the stopping criterion is satisfied.

### 2.4. *q*-Thresholding Algorithm

*q*-thresholding is a joint recovery algorithm proposed in [36]. If the joint sparsity level *K* is known, we can estimate the joint support set of joint signal by using the following:

(12) I^Φ,Y,K,q=indices  of  the  K  largest  values  in  Φ:,jTYqj=1n

## 3. DCS-GWO: Grey Wolf Optimizer Algorithm for Distributed Compressed Sensing

In this part, we firstly introduce the DCS-GWO in Section 3.1. Next, we analyze DCS-GWO's computational complexity in Section 3.2.

### 3.1. DCS-GWO

DCS-GWO is essentially a GWO algorithm. It has four basic elements: cost function, initial positions, generating mechanism, and stopping criterions. In this part, we firstly introduce DCS-GWO's four basic elements and then summarize it in Algorithm 1.

*(1) Cost Function. *Similar to DC-SAMP and DCS-FBP, we can use a two-step strategy to solve the DCS problem. Firstly, the joint support set *I* of **X** is estimated. Then, the joint signal can be estimated by using the least square method as follows:

(13) rec XI^,:=Φ:,I^†Y,rec XS−I^,:=0,

where $\hat{I}$ denotes the estimated joint support set and *S*≜{1, 2,…, *n*}.

We can find that if the joint support set is estimated accurately, it must satisfy ${\left\|\Phi_{:,\hat{I}}\Phi_{:,\hat{I}}^{†}Y-Y\right\|}_{F}=0$. As ${\left\|\Phi_{:,\hat{I}}\Phi_{:,\hat{I}}^{†}Y-Y\right\|}_{F}\geq 0$, we define the cost function as

(14) $$\begin{array}{c} f\left(\;I\;\right)={\left\|\;\Phi_{\text{:},\hat{I}}\Phi_{\text{:},\hat{I}}^{†}Y-Y\;\right\|}_{F}. \end{array}$$

We can estimate the joint support set by solving the following:

(15) minI∈Θ fI,

where Θ is the set consisting of all the *K*-cardinality subsets of *S*.

*(2) Initial Positions. *We assume that the wolf number set *L*_wolf_ = {1,2,…, *L*}. We use the *q*-thresholding algorithm to initialize the grey wolves' positions. *I*_*l*_^0^ denotes the initial position of the *l*th wolf where *l* ∈ *L*_wolf_. It is estimated by using

(16) $$\begin{array}{c} I_{l}^{0}≜\hat{I}\left\{\;\Phi ,Y,K,q_{l}\;\right\},\;l\in L_{wolf}, \end{array}$$

where *q*_*l*_ is a random number in the closed interval [1,2].

*(3) Update Strategy. *The update strategy of DCS-GWO inherits from GWO. In DCS-GWO, *t* denotes the current iteration. For *l* ∈ *L*_wolf_, the *l*th wolf's position is updated according to the previous best three wolves' positions *I*_best1_^*t*−1^, *I*_best2_^*t*−1^, and *I*_best3_^*t*−1^ and its previous position *I*_*l*_^*t*−1^. A parameter *C*_*n*_ is used to limit the set size where *K* < *C*_*n*_ < spark(Φ).

The position of the *l*th wolf is updated as follows. In Step 1.1, a set *U*_1_ is formed according to the previous best three positions and the *l*th wolf's previous position, *U*_1_ = (*I*_best1_^*t*−1^∩*I*_best2_^*t*−1^∩*I*_best3_^*t*−1^) ∪ *I*_*l*_^*t*−1^. In Step 1.2, if |*U*_1_| > *C*_*n*_, randomly choose *C*_*n*_ − (*I*_best1_^*t*−1^∩*I*_best2_^*t*−1^∩*I*_best3_^*t*−1^) elements from *I*_*l*_^*t*−1^ to form the set *U*_2_ and define the temporary position *U* = (*I*_best1_^*t*−1^∩*I*_best2_^*t*−1^∩*I*_best3_^*t*−1^) ∪ *U*_2_; if |*U*_1_| < *C*_*n*_, randomly choose *C*_*n*_ − |*U*_1_| elements from *S* − *U*_1_ to form a set *U*_3_ and define the temporary position *U* = *U*_1_ ∪ *U*_3_. In Step 1.3, the temporary solution rec **X**_*l*_^*t*^ is estimated by using the least square method as

(17) rec XltU,:=Φ:,U†Y,rec XltS−U,:=0.

Lastly, the position of the *l*th wolf is updated by

(18) Ilt≜indices  of  the  K  largest  values  in  rec Xlti,:qi=1n,

where *l* ∈ {1,2,…, *L*}. After all wolves' positions are updated, we can update the best three wolves' positions according to the cost function.

*(4) Stopping Criterion. *In order to avoid too many iterations, we set a maximum allowed iteration number *N*_max_ and a small positive number *ε* as stopping criterions. If *f*(*I*_best1_^*t*^) < *ε* or the number of iterations reaches *N*_max_, the iteration process is terminated.

We summarize the DCS-GWO in Algorithm 1.

### 3.2. Computational Complexity Analysis of DCS-GWO

According to Algorithm 1, the initialization and iteration contribute the main computational complexity of DCS-GWO. The computational complexity of initialization is *O*(*LJmn*). In each iteration, the main computational complexity lies in Steps 1.3 and 1.4 which, respectively, have upper limit value *O*(*m*^3^) + *O*(*Jm*^2^) and *O*(*mK*^2^) + *O*(*JmK*). Because *K* ≤ *m*, the computational complexity upper limit value of each iteration is *O*(*m*^3^) + *O*(*Jm*^2^). Because the total number of iterations is not more than *LN*_max_, the computational complexity upper limit value of DCS-GWO is *O*(*N*_max_*Lm*^3^) + *O*(*N*_max_*LJm*^2^) + *O*(*LJmn*). Obviously, as an intelligent optimizer algorithm, DCS-GWO has higher computational complexity than greedy pursuit algorithms. However, as swarm intelligence algorithm, it can run in parallel to reduce the running time.

## 4. Simulation Results and Analysis

### 4.1. Experiment Configuration

In this section, the performance of DCS-GWO is compared with other algorithms that can solve the DCS problem based on JSM-3, including Joint OMP [20], Joint SP [20], DCS-SAMP [21], and DCS-FBP [22]. The algorithms proposed in [15–18] are designed for JSM-1 or JSM-2, they are not discussed in this part. The parameters of DCS-GWO are set as *N*_max_ = max(10*∗K*, 500), *L* = 8, *C*_*n*_ = 0.8 m, and *ε* = 1*e* − 5. We use the following hypothesis in the simulation.

We use the Gaussian random matrix as measurement matrix Φ, the elements of which are randomly drawn from the standard i.i.d. and every column of which is normalized to unit *l*_2_ norm. All signals follow the JSM-3 with the common sparsity *K*_*c*_ and innovation sparsity *K*_*j*_. We assume that the innovation sparsity *K*_*j*_ is the same for all signals. For each signal, the support sets of common component and innovation component are random subsets of the set *S* = {1,2,…, *n*}. The nonzero coefficients of the common component and innovation component are randomly drawn from the standard i.i.d. In each experiment, 200 independent trials are conducted. In each trial, the signals and measurement matrix Φ are generated independently. Average Normalized Mean Squared Error (ANMSE), perfect recovery percentage, and average runtime are used to evaluate the algorithms. The ANMSE is defined as

(19) ANMSE=1200∑i=120010×logXi−X^i22Xi22.

The perfect recovery condition is ${\left\|X^{\left(i\right)}-{\hat{X}}^{\left(i\right)}\right\|}_{2}<10^{-2}{\left\|X^{\left(i\right)}\right\|}_{2}$, where **X**^(*i*)^ and ${\hat{X}}^{\left(i\right)}$, respectively, denote the original joint signal and the recovered joint signal in the *i*th trial. If *N*_*S*_ trials are success, the perfect recovery percentage is *N*_*S*_/200. All the experiments are implemented by using Matlab R2014a on the computer with 2.5 GHz Intel Core I3 processor and 4.0 GB memory running window 7 system.

### 4.2. Experiment Results

*(1) Requirement for Measurements. *In the first simulation, we compare the performance of all algorithms against the measurement number *M* changing from 50 to 90 with Step 10. Other parameters are fixed as *N* = 256, *K*_*c*_ = 15, *K*_*j*_ = 5, and *J* = 3.

**Figure 1:**
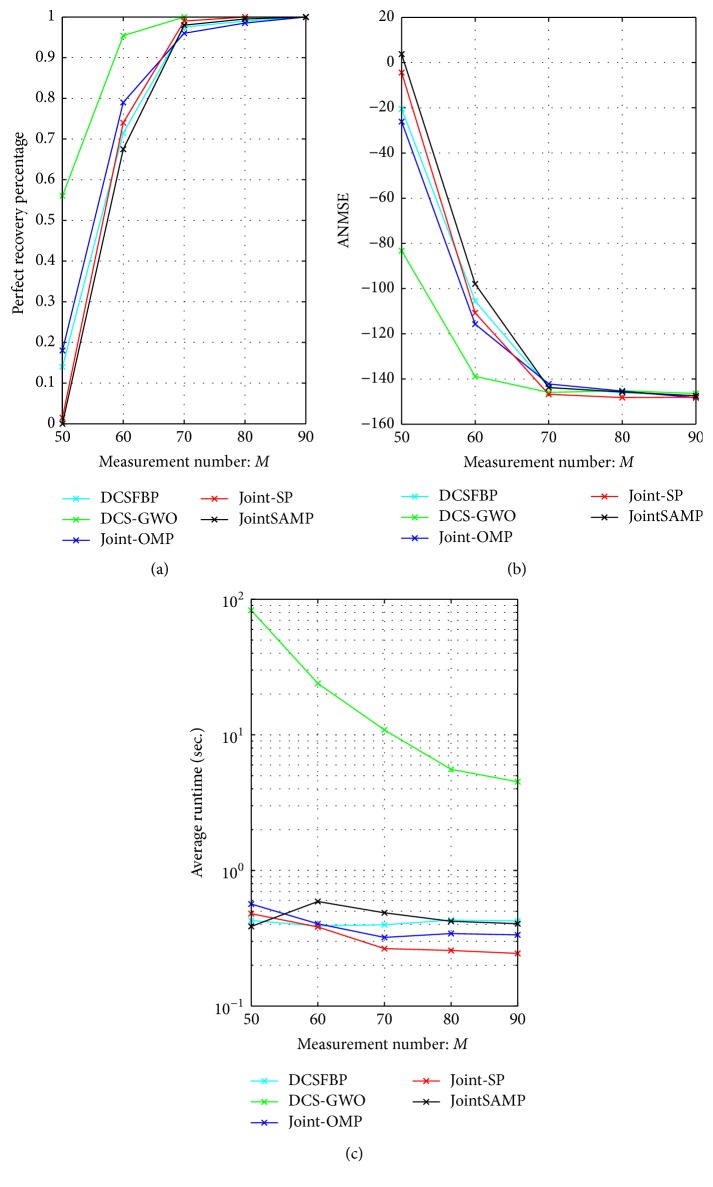
Recovery performance of different algorithms against *M* with *N* = 256, *K*_*c*_ = 15, *K*_*j*_ = 5, and *J* = 3. (a) Perfect recovery percentage. (b) ANMSE. (c) Average runtime.

As Figure 1(a) shows that the perfect recovery percentage of DCS-GWO is always higher than other algorithms. Besides, DCS-GWO needs less measurement number to perfectly recover signals. When the measurement number reaches 70, DCS-GWO recovers signals perfectly. Other algorithms need the measurement number 90 to perfectly recover signals. As Figure 1(b) shows, DCS-GWO has lower ANMSE than other algorithms.

From Figure 1(c), at the expense of global search ability, DCS-GWO needs more running time than other algorithms. However, as a swarm intelligence algorithm, it can run in parallel to reduce the running time.

*(2) Robustness against Common Sparsity. *In the second simulation, we evaluate the performance of the algorithms against the common sparsity *K*_*c*_ changing from 1 to 15 with the step size 2. Other parameters are fixed as *N* = 256, *M* = 40, *J* = 3, and *K*_*j*_ = 3.

**Figure 2:**
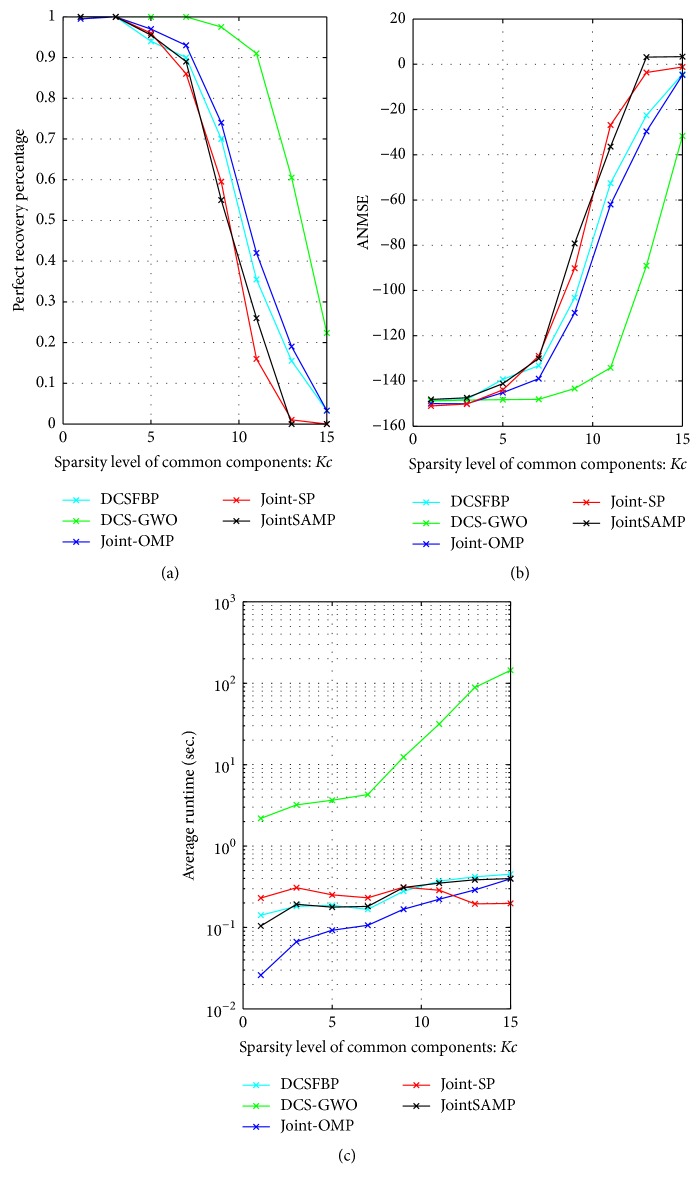
Recovery performance of different algorithms against *K*_*c*_ with *N* = 256, *M* = 40, *K*_*j*_ = 3, and *J* = 3. (a) Perfect recovery percentage. (b) ANMSE. (c) Average Runtime.

As Figure 2(a) shows, DCS-GWO performs far better than other algorithms in the perfect recovery percentage. For all algorithms, as the common signal sparsity *K*_*c*_ increases, the perfect recovery percentage decreases. We are more interested in at which sparsity the perfect recovery percentages drop below 1. The perfect recovery percentage of DCS-GWO starts to fall below 1 when *K*_*c*_ > 7; however, other algorithms already fall below 1 when *K*_*c*_ > 3. From Figure 2(b), DCS-GWO has lower ANMSE than other algorithms. As for average runtime, we can get the similarly results as Figure 1(c).

*(3) Robustness against Innovation Sparsity. *In the third simulation, we compare the performance of DCS-GWO with other algorithms against the innovation sparsity *K*_*j*_ changing from 0 to 7 with Step 1. Other parameters are set as *N* = 256, *M* = 40, *J* = 3, and *K*_*c*_ = 5.

**Figure 3:**
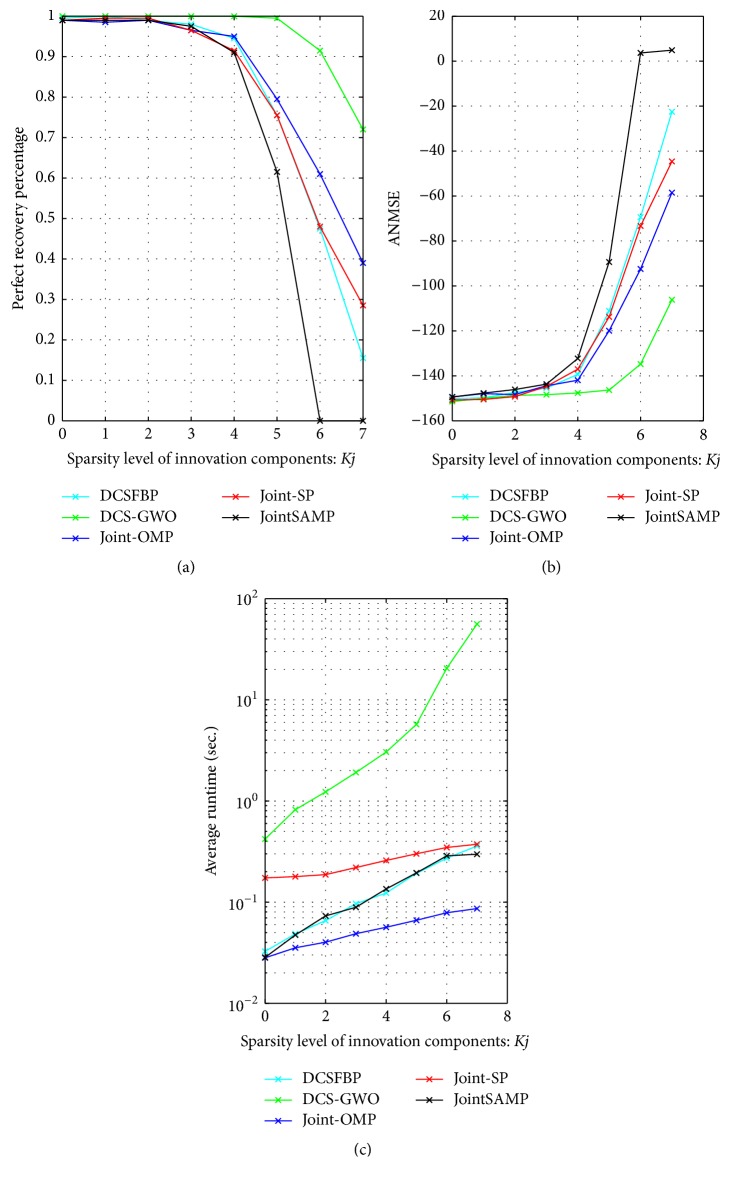
Recovery performance of different algorithms against *K*_*j*_ with *N* = 256, *M* = 40, *K*_*c*_ = 5, and *J* = 3. (a) Perfect recovery percentage. (b) ANMSE. (c) Average runtime.

As Figure 3(a) shows, the DCS-GWO performs extremely better than other algorithms in perfect recovery percentage. As the increase of the innovation sparsity *K*_*j*_, the perfect recovery percentage is declining, that is, because the increase of joint sparsity level influences the performance of all algorithms. The perfect recovery percentages of other algorithms start to fall below 1 when *K*_*j*_ > 2; our algorithm starts to fall below 1 until *K*_*j*_ > 5. As in Figure 3(b), DCS-GWO has lower ANMSE than other algorithms. As for average runtime, we can get the similar results as Figure 1(c).

*(4) Robustness against the Number of Signals. *We compare our algorithm with other algorithms against the number of signals *J* changing from 2 to 6 with Step 1. Other parameters are set as *N* = 256, *M* = 40, *K*_*c*_ = 4, and *K*_*j*_ = 4.

The perfect recovery percentages of Joint SP and Joint OMP are not influenced obviously by the increase of signals number, because both of them recover the signals one by one rather than jointly. They have better performance than our algorithms when *J* > 5; however, our algorithm performs better than them when *J* ≤ 5.

**Figure 4:**
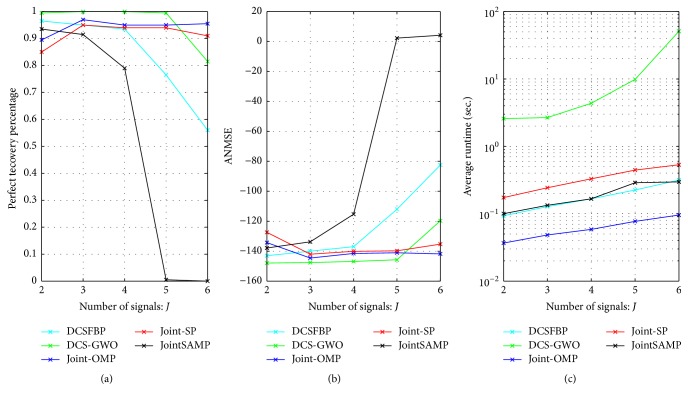
Recovery performance of different algorithms against *J* with *N* = 256, *M* = 40, *K*_*c*_ = 4, and *K*_*j*_ = 4. (a) Perfect recovery percentage. (b) ANMSE. (c) Average runtime.

As the number of signals *J* increases, the perfect recovery percentages of DCSFBP, DCS-GWO, and DCSSAMP decrease. As Figure 4(a) shows, our algorithm has higher perfect recovery percentages than DCSFBP and DCSSAMP. When *J* > 2, the perfect recovery percentages of DCS-FBP and DCSSAMP already fall below 1; however, our algorithm starts to fall below 1 until *J* > 5. As Figure 4(b), DCS-GWO has lower ANMSE than other algorithms. As for average runtime, we can get the similarly results as Figure 1(c).

From the above simulations, we can see that DCS-GWO has higher perfect recovery percentage and lower ANMSE than greedy pursuit algorithms. Next, we analyze the reason of DCS-GWO's better performance according to its structure.

The main reason for DCS-GWO's better performance is its update mechanism. In Step 1.1, the common information of the best three grey wolves' positions is utilized to update all the grey wolves' positions. By this way, the previous obtained best information is preserved in the new grey wolves' positions. In Step 1.2, random perturbations are introduced into the grey wolves' positions. Benefitting from this, DCS-GWO can skip the local optimum position and guide the search in promising directions. In Steps 1.3 and 1.4, *C*_*n*_ − *K* indices corresponding to the rows of the temporal joint signal which have smallest row norm values are removed from the temporal support set. By this way, the previous wrong selected indices can be removed from the temporal support set.

In contrast to DCS-GWO, greedy pursuit algorithms search the support set according to the gradient of (15), which is a local optimal search mechanism. Therefore, due to the efficiency of DCS-GWO's update mechanism, DCS-GWO can find the support set more accurately than greedy pursuit algorithms. Then, DCS-GWO can recover the signals more accurately than greedy pursuit algorithms.

## 5. Conclusion

In this paper, an intelligent grey wolf optimizer algorithm is proposed to solve the DCS problem based on JSM-3. The positions of grey wolves are initialized by using the *q*-thresholding algorithm and updated by using the idea of GWO. Inheriting the global search ability of GWO, DCS-GWO overcomes greedy pursuit algorithms' shortcoming of easily falling into suboptimal solutions. The simulation results illustrate that DCS-GWO has higher perfect recovery percentage and lower ANMSE than other algorithms. DCS-GWO has higher computational complexity than other algorithms. However, as a swarm intelligence algorithm, it can compute in parallel to reducing the running time. In the future work, we will focus on developing effective update strategies to reduce the running time. Moreover, there are many more recent nature-inspired algorithms, such as Ant Lion Optimizer (ALO) [37], Moth-Flame Optimization (MFO) algorithm [38], Whale Optimization Algorithm (WOA) [39], and Multiverse Optimizer (MVO) [40]. We will exploit them to solve the DCS problem based on JSM-3.

## Figures and Tables

**Algorithm 1:**
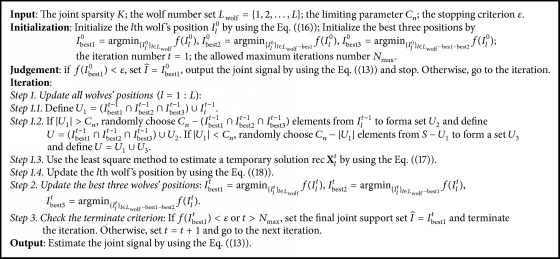
DCS-GWO.
